# Noninvasive prognostication of hepatocellular carcinoma based on cell-free DNA methylation

**DOI:** 10.1371/journal.pone.0321736

**Published:** 2025-04-25

**Authors:** Ran Hu, Benjamin Tran, Shuo Li, Mary L. Stackpole, Weihua Zeng, Yonggang Zhou, Andrew Melehy, Saeed Sadeghi, Richard S. Finn, Xianghong Jasmine Zhou, Wenyuan Li, Vatche G. Agopian

**Affiliations:** 1 Department of Pathology and Laboratory Medicine, David Geffen School of Medicine, University of California at Los Angeles, Los Angeles, California, United States of America; 2 Bioinformatics Interdepartmental Graduate Program, University of California at Los Angeles, Los Angeles, California, United States of America; 3 Institute for Quantitative and Computational Biosciences, University of California at Los Angeles, Los Angeles, California, United States of America; 4 Department of Surgery, David Geffen School of Medicine, University of California at Los Angeles, Los Angeles, California, United States of America; 5 Department of Medicine, Division of Hematology/Oncology, David Geffen School of Medicine, University of California at Los Angeles, Los Angeles, California, United States of America; 6 Jonsson Comprehensive Cancer Center, University of California at Los Angeles, Los Angeles, California, United States of America; University of Navarra School of Medicine and Center for Applied Medical Research (CIMA), SPAIN

## Abstract

**Background:**

The current noninvasive prognostic evaluation methods for hepatocellular carcinoma (HCC), which are largely reliant on radiographic imaging features and serum biomarkers such as alpha-fetoprotein (AFP), have limited effectiveness in discriminating patient outcomes. Identification of new prognostic biomarkers is a critical unmet need to improve treatment decision-making. Epigenetic changes in cell-free DNA (cfDNA) have shown promise in early cancer diagnosis and prognosis. Thus, we aim to evaluate the potential of cfDNA methylation as a noninvasive predictor for prognostication in patients with active, radiographically viable HCC.

**Methods:**

Using Illumina HumanMethylation450 array data of 377 HCC tumors and 50 adjacent normal tissues obtained from The Cancer Genome Atlas (TCGA), we identified 158 HCC-related DNA methylation markers associated with overall survival (OS). This signature was further validated in 29 HCC tumor tissue samples. Subsequently, we applied the signature to an independent cohort of 52 patients with plasma cfDNA samples by calculating the cfDNA methylation-based risk score (methRisk) via random survival forest models with 10-fold cross-validation for the prognostication of OS.

**Results:**

The cfDNA-based methRisk showed strong discriminatory power when evaluated as a single predictor for OS (3-year AUC = 0.81, 95% CI: 0.68–0.94). Integrating the methRisk with existing risk indices like Barcelona clinic liver cancer (BCLC) staging significantly improved the noninvasive prognostic assessments for OS (3-year AUC = 0.91, 95% CI: 0.80–1), and methRisk remained an independent predictor of survival in the multivariate Cox model (P = 0.007).

**Conclusions:**

Our study serves as a pilot study demonstrating that cfDNA methylation biomarkers assessed from a peripheral blood draw can stratify HCC patients into clinically meaningful risk groups. These findings indicate that cfDNA methylation is a promising noninvasive prognostic biomarker for HCC, providing a proof-of-concept for its potential clinical utility and laying the groundwork for broader applications.

## Introduction

Hepatocellular carcinoma (HCC) is a highly heterogeneous malignancy and the most prevalent type of primary liver cancer that arises predominantly in patients with cirrhosis. Various etiological factors contribute to the initiation and progression of HCC, including hepatitis B virus (HBV) and hepatitis C virus (HCV) infection, alcohol use disorder, and metabolic dysfunction-associated steatotic liver disease (MASLD), previously referred to as nonalcoholic fatty liver disease. In patients with early stage HCC, surgical resection is widely accepted as the gold standard curative therapy for those with a limited tumor burden and without prohibitive portal hypertension; while liver transplantation is reserved for patients with unresectable HCC meeting specific criteria [1]. Despite the current clinical-radiologic staging systems and serum biomarkers, the substantial inter- and intra-tumor heterogeneity in HCC obscures the underlying tumor biology, presenting significant challenges in predicting recurrence and survival. While alpha-fetoprotein (AFP) is currently the only blood-based biomarker adopted for HCC prognostic assessment, nearly 40% of tumors do not secrete AFP, limiting its utility [2]. Pathologic tumor features such as grade and differentiation can augment prognostication in addition to imaging and AFP; however, presurgical percutaneous biopsies are not routinely pursued, require invasive procedures, and may have limited sensitivity in detecting the true pathologic features [3], reducing their utility for clinical decision making. Therefore, there is a critical unmet need to develop noninvasive blood-based HCC biomarkers that can accurately stratify patients into clinically meaningful risk groups and guide therapeutic decisions.

Aberrant DNA methylation changes occur early in tumorigenesis and continue to accumulate during tumor progression [4], making them a promising indicator of tumor recurrence and overall survival [5]. Previous studies have revealed the prognostic potential of DNA methylation profiles from liver tissue specimens of patients with HCC. Villanueva et al. identified a signature of 36 DNA methylation markers that predicted poor survival in patients with HCC and validated the signature using tumor tissue samples [6]. Hernandez-Meza et al. reported that DNA methylation profiles from tumor-adjacent cirrhotic tissues were associated with clinical outcomes [7]. Recently, circulating cell-free DNA (cfDNA) has emerged as a noninvasive surrogate for cancer diagnosis and prognosis, as it can provide valuable information about tumor-specific genetic and epigenetic alterations and monitor treatment response and disease progression. While some studies have explored the use of small panels of cfDNA methylation biomarkers for HCC prognosis [8,9], these panels are insufficient to sensitively capture reliable cancer signals, as they do not adequately reflect the complex and heterogeneous nature of epigenetic aberrations. cfDNA methylation aberrations vary across different cancer subtypes, stages, and etiologies, highlighting the need for broader and more comprehensive biomarker panels to effectively encompass the full spectrum of epigenetic alterations associated with HCC.

In this study, we aim to identify a large panel of cfDNA methylation markers to explore a more effective ***noninvasive approach*** for HCC prognosis. Using the Illumina HumanMethylation450 array data of 377 HCC tumors and 50 adjacent normal liver tissues from The Cancer Genome Atlas (TCGA), we identified 158 DNA methylation markers that are predictive of survival. We validated these markers using the Reduced Representation Bisulfite Sequencing (RRBS) data of HCC tumor tissue samples from 29 surgically resected patients. Functional analyses of these markers indicated their roles in regulating gene expression and revealed the biological pathways associated with HCC patient survival. To evaluate the noninvasive prognostic value of these markers, we developed a cfDNA methylation-based overall survival (OS) risk score, referred to as “methRisk”, by employing random survival forest (RSF) models on these markers with plasma cfDNA methylation data. To assess the methRisk in a preliminary application, we collected plasma cfDNA samples from an independent cohort of 52 HCC patients, and profiled the methylation patterns of these samples using our newly developed cost-effective cfDNA methylome sequencing assay, cfMethyl-Seq [10]. We performed 10-fold cross-validation in this cohort to evaluate the prognostic performance of the methRisk. The methRisk effectively stratified patients into statistically distinct risk groups as a single predictor. Integration of the methRisk with risk indices such as Barcelona clinic liver cancer (BCLC) exhibited enhanced performance, highlighting its potential to improve therapeutic decision-making.

## Methods

### Human samples

The study enrolled 29 HCC patients with surgically resected tumor tissue samples (S1 Table) and an additional 52 HCC patients with plasma samples (Table 1), collected from the Ronald Reagan UCLA Medical Center. This study was approved by the institutional review board (IRB# 14-001932) of the University of California at Los Angeles, and was conducted in accordance with the ethical regulations of the Declaration of Helsinki and Istanbul. All the participants provided written informed consent. Clinical data for this study was last accessed in October 2023 for research purposes. BT, AM, and VGA had access to information that could identify individual participants during or after data collection. We collected detailed clinical information of all participants, including age, sex, etiology, radiological tumor features (including largest tumor size and tumor number), AFP levels measured in close proximity to the collection of plasma cfDNA (prior AFP), MELD (model for end-stage liver disease) score, BCLC staging, transplant criteria, locoregional treatment, therapy, and survival outcomes. Transplant criteria were categorized as 1 if within Milan, 2 if beyond Milan but within UCSF criteria, 3 if locally advanced beyond UCSF criteria but without metastases, and 4 if there was extrahepatic metastasis. Note that the MELD score is only available to cirrhotic patients, resulting in missing data within both cohorts. All 52 plasma samples were collected from patients with active radiographically viable HCC tumors at the time of enrollment. Of these, 36 samples were collected prior to the administration of any locoregional treatment or systematic therapy, while the remaining 16 patients had undergone some treatment years ago but had developed a new viable tumor. The follow-up time was calculated from the date of blood draw until the last known follow-up or death.

**Table 1:** Characteristics of 52 HCC patients with plasma samples.

Characteristic	No.
Median age (IQR)	66.5 (60.8, 72.0)
Sex, male (%)	39 (75%)
Etiology (%)	
HCV	23 (44.2%)
HBV	7 (13.5%)
MASH	15 (28.8%)
ALD	3 (5.8%)
Others	4 (7.7%)
Median largest tumor size, cm (IQR)	3.4 (2.2, 6.4)
Multiple nodules present (%)	15 (28.8%)
Median prior AFP, ng/ml (IQR)	10.3 (5.0, 29.1)
Median MELD score (IQR)	8 (6, 9)
BCLC stage (%)	
0	2 (3.8%)
A	33 (63.5%)
B	6 (11.5%)
C	11 (21.2%)
Transplant criteria	
1	29 (55.8%)
2	5 (9.6%)
3	13 (25%)
4	5 (9.6%)
LRT and/or ST before sample collection, yes (%)	16 (30.8%)
LRT and/or ST after sample collection, yes (%)	21 (40.4%)
Events (%)	
Death	15 (28.8%)
Recurrence or progression	31 (59.6%)
Median follow-up, months	44

AFP, alpha-fetoprotein; ALD, alcoholic liver disease; BCLC, Barcelona Clinic Liver Cancer; HBV, hepatitis B virus; HCV, hepatitis C virus; IQR, interquartile range; LRT, locoregional treatment; MASH, metabolic dysfunction-associated steatohepatitis; MELD, model for end-stage liver disease; ST, systemic therapy.

Genomic DNA was extracted from 10–100 mg of solid tissue samples using QIAGEN blood and tissue kits, followed by sequencing using RRBS. For cfDNA extraction, 1–4 ml of plasma was used with QIAGEN QIAamp circulating nucleic acid kits (Catalog# 55114, Germantown, MD) according to the manufacturer’s protocol. The genome-wide methylation of cfDNA was profiled using the cfMethyl-Seq assay [10]. Detailed information on the data preprocessing steps is provided in the S1 File. Raw sequencing data were produced in our previous study [10] and have been deposited in the European Genome-phenome Archive (EGA) under the accession code EGAS00001006020. Access to the data requires approval from the relevant data access agreement.

### Data analysis

To analyze the Illumina 450K methylation array data of 377 HCC tumors and 50 adjacent normal liver tissue samples from TCGA, we first performed normalization using the *preprocessFunnorm* function in the R package *minfi* [11]. Subsequently, we filtered out probes that (1) had a detection p-value > 0.01 in one or more samples; (2) mapped to sex chromosomes; (3) contained single-nucleotide polymorphisms (SNPs); and (4) mapped to multiple positions in the genome [12]. Following preprocessing, 369,561 probes remained available for analysis.

**Fig 1:**
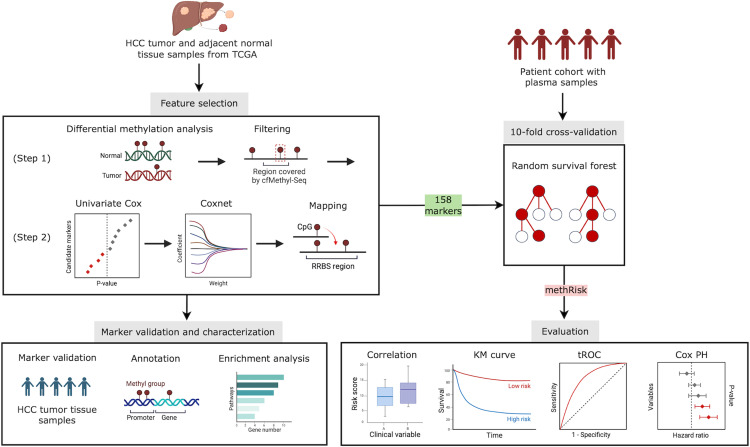
Schematic diagram of the study design. We utilized the TCGA dataset in a two-step feature selection process to identify 158 methylation markers that are associated with HCC and survival. We validated these markers’ prognostic value in our HCC tumor tissue samples. We then applied random survival forest with 10-fold cross-validation to predict the cfDNA methylation-based overall survival risk score (methRisk) for plasma samples of an independent patient cohort. Subsequent functional analysis and various metrics were used to evaluate the performance of the methRisk. Figure created with BioRender.com.

The discovery of prognostic methylation markers was conducted in two primary steps utilizing the methylation array data from the TCGA Cohort (Fig 1): (Step 1) the identification of HCC-specific tumor markers through differential methylation analysis between tumor and normal tissues; (Step 2) further selection of survival-related markers from those identified in Step 1, using univariate Cox analysis and Coxnet models on tumor samples with survival information.

In the first step of identifying HCC-specific tumor markers, we performed a probe-wise differential methylation analysis using the R package *limma* [13] on the M-values [14] between 50 pairs of HCC tumor and adjacent normal tissue samples (paired test), as well as between these 50 normal tissues and the remaining 327 HCC tumor tissues (unpaired test). The M-value, which represents the log2 ratio of the intensities between methylated and unmethylated probes, was used here because of its statistical validity over the Beta-value in differential methylation analysis [14]. Our analysis revealed 128,955 differentially methylated probes (DMPs) that were consistently identified across both paired and unpaired tests, with each DMP (or CpG site) demonstrating a Benjamini-Hochberg adjusted p-value < 0.05. In light of the intended use of these markers for noninvasive prognosis based on cfMethyl-Seq data of cfDNA, we performed an additional filtration step to exclude DMPs that were not effectively covered in the cfMethyl-Seq assay, resulting in a final selection of 46,665 DMPs.

In the second step, we leveraged the survival data of the TCGA samples to identify candidate CpG sites associated with OS [15]. First, we performed univariate Cox proportional hazards regression [16] analysis on the Beta-values of CpG sites selected in Step 1 and kept 5,948 sites that passed the Wald tests (P < 0.05). The Beta-value for each CpG site was computed as the ratio of intensity from methylated probes to the total intensity from all probes targeting the same site for the 450K array data, or the ratio of methylated sites to all sites on reads mapped to the CpG site for the RRBS data. Subsequently, we utilized the multivariate Cox model regularized by elastic net penalties (Coxnet) [17] and identified a panel of 159 candidate CpG sites that had non-zero coefficients in at least five out of ten rounds of 5-fold cross-validation. To ensure more robust methylation signals from low-coverage RRBS and cfMethyl-Seq data and by exploiting the pervasive nature of DNA methylation, we used the average Beta-value of all CpG sites within RRBS genomic regions as markers. The RRBS genomic regions were defined as the genomic locations between two adjacent MspI restriction enzyme digestion sites (i.e., between two CCGG sites) and that are less than 350 base pairs. The final marker panel was comprised of 158 RRBS regions that correspond to the identified CpG sites (S2 Table).

We visualized the methylation status of markers highly correlated with survival in the patient cohort with tissue and plasma samples using heatmaps (S1 Fig). To validate the DNA methylation marker panel in our tissue samples (n = 29), we performed survival analysis on the RRBS data of these samples. To assess the noninvasive predictive capability of the markers in the plasma samples (n = 52), we built and evaluated prognostic models with 10-fold cross-validation (Fig 1). Specifically, we partitioned the plasma samples into ten folds by stratified sampling based on the censoring rate. We then employed the RSF algorithm to train a prognostic model on nine folds (training set) and predict the risk scores for the remaining fold (validation set). We repeated this training-validation process ten times and combined the resulting risk scores of all samples into a list. The list of risk scores enabled the stratification of patients into low- and high-risk groups and was evaluated using various metrics.

## Results

### Validating the prognostic DNA methylation markers in HCC tumor tissue samples

We validated the DNA methylation markers in our 29 HCC tumor tissue samples by performing a survival analysis and compared it with each clinical/radiological feature. In the survival analysis, risk scores were predicted using 10-fold cross-validation and RSF models applied to 158 DNA methylation markers. The risk scores demonstrated superior performance, achieving the highest area under the time-dependent receiver operating characteristic curve (time-dependent AUC) (3-year AUC = 0.87, 95% CI: 0.72–1; S2 Fig) compared to prior AFP, radiological features (i.e., largest tumor size and tumor number), and MELD score. The results showed that these markers identified from the TCGA dataset carry the prognostic signals in the HCC tissue samples.

### Functional analysis of the prognostic DNA methylation markers

**Fig 2:**
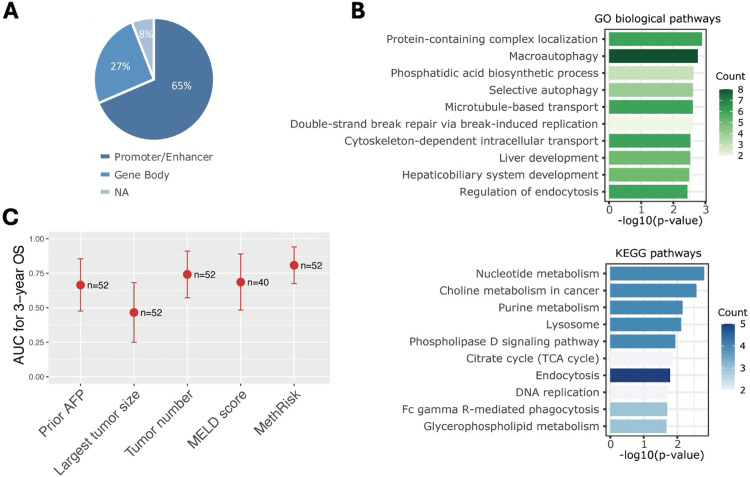
Functional analysis of DNA methylation markers and cfDNA-based methRisk performance. **(A)** Composition of markers in terms of genome annotation. **(B)** KEGG pathway enrichment analysis, and GO enrichment analysis of biological pathways associated with the methylation marker-related genes. The bar charts show the top 10 enriched terms ranked by p-value. **(C)** Time-dependent AUCs for individual features predicting 3-year survival in the patient cohort with plasma samples. Error bars represent the 95% confidence intervals. The sample size (n) is annotated to the right of the error bar.

To annotate the DNA methylation markers, we employed databases including GeneHancer [18], GENCODE (version 19) [19], and DNMIVD [20] to map the RRBS regions to genomic features (i.e., promoters, enhancers and gene bodies). Among the 158 markers, 65% were found to be located in the promoter or enhancer region (Fig 2A), indicating their potential role in regulating gene transcription.

To gain deeper insight into the biological functions associated with the marker panel, we conducted comprehensive pathway and functional enrichment analyses. Specifically, we employed Kyoto Encyclopedia of Genes and Genomes (KEGG) [21] pathway analysis and Gene Ontology (GO) [22] enrichment analysis (Fig 2B) using the R package *clusterProfiler* [23]. KEGG analysis revealed the enrichment of pathways crucial for regulating cellular processes and closely linked to the hallmarks of cancer. Pathways that are essential for cancer cell growth and proliferation including nucleotide metabolism, purine metabolism, and DNA replication were involved. Choline metabolism in cancer was enriched and has been suggested to play a role in the development and progression of HCC [24]. The analysis revealed the involvement of an important metabolic pathway, citrate cycle (TCA cycle), which has been shown to be downregulated in HCC [25]. Moreover, disruptions in the lysosome or endocytosis process have been suggested to contribute to cancer development and may be linked to poor prognosis [26]. Phospholipase D signaling pathway [27] and glycerophospholipid metabolism [28] were involved and have been shown to be related to tumorigenesis and cancer progression. It also revealed the involvement of Fc gamma R-mediated phagocytosis [29], a process known for its role in mediating tumor cell killing. GO analysis of biological pathways illuminated biological processes related to liver cancer progression and prognosis. Pathways involved in intracellular protein trafficking, such as protein-containing complex localization [30], microtubule-based transport [31], and cytoskeleton-dependent intracellular transport [32], have been suggested to correlate with poor survival in cancer. Furthermore, enriched pathways, including macroautophagy [33], phosphatidic acid biosynthetic process [34], selective autophagy [35], and regulation of endocytosis [36] are commonly dysregulated in cancer. Additionally, double-strand break repair via break-induced replication [37] was included, which may play a crucial role in preventing the accumulation of mutations that contribute to the onset and progression of cancer.

### Validation of the prognostic DNA methylation markers through transcriptomics analyses

We further validated the prognostic significance of protein-coding genes associated with our marker panel using the Human Pathology Atlas from the Human Protein Atlas program [38] (data available from v22.proteinatlas.org/about/download). Among the methylation marker-related genes that underwent survival analysis on mRNA expression data in HCC patients, 65% genes had a log-rank test p-value < 0.05 (S3 Table), suggesting that these genes might play an important role in affecting survival rates. The most significant prognostic gene with the lowest p-value was EIF2B5 (P = 3.38 × 10^−8^), which was suggested to be associated with poor prognosis of HCC patients [39]. Other genes that exhibited prognostic potential in HCC include RNF34 (P = 6.72 × 10^−8^), SKA1 (P = 9.60 × 10^−8^), SLC16A3/MCT4 (P = 1.19 × 10^−7^), SLC25A19 (P = 1.21 × 10^−7^), BRD9 (P = 5.88 × 10^−7^), and CIT (P = 3.61 × 10^−6^) [40–45]. Although some genes have not been well studied in HCC, they have been described in other cancer types. For instance, overexpression of SLC35G2/TMEM22 (P = 1.16 × 10^−5^) has been linked to the progression of clear cell renal cell carcinoma [46]. In addition, genes with less significant log-rank test p-values may play potential roles in tumorigenesis. For example, PARP3 (P = 0.4128) has been reported to contribute to tumor progression [47], and NR2F2 (P = 0.3383) has been identified as a critical regulator of malignant cancer stem cell functions [48].

### Evaluation of the noninvasive prognostic value of the DNA methylation markers in HCC plasma samples

Using plasma samples, we obtained cfDNA methylation profiles for the marker panel and calculated the cfDNA-based methRisk scores. We compared the time-dependent AUCs of the methRisk with clinical/radiological features in the patient cohort with plasma samples. The methRisk predicted from the cfDNA methylation data exhibited the highest predictive accuracy, achieving a 3-year AUC of 0.81 (95% CI = 0.68–0.94; Fig 2C), surpassing the clinical and radiological features.

**Fig 3:**
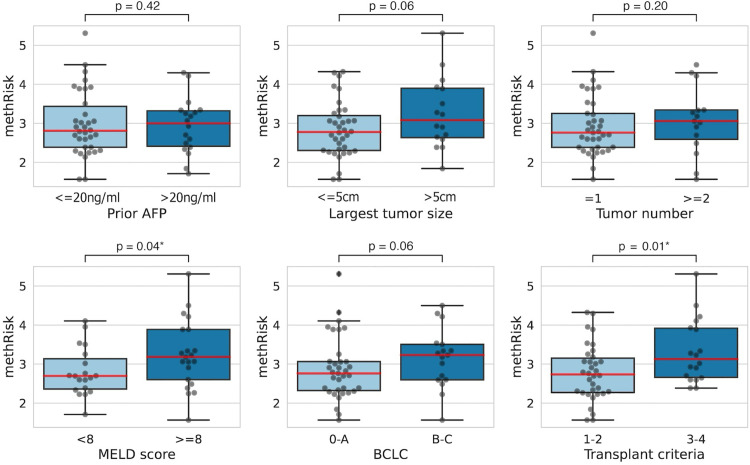
Box plots illustrating the methRisk distribution among HCC patient subgroups in the patient cohort with plasma samples. Patients were stratified based on prior AFP, largest tumor size, tumor number, MELD score, BCLC, and transplant criteria. P-values from the one-sided Mann-Whitney-Wilcoxon test comparing methRisk between subgroups are indicated above (* P < 0.05). The median methRisk value of each patient subgroup is denoted by a red line.

Using this cohort, we further investigated the associations between the methRisk and each known clinical/radiological risk factor related to poor prognosis. A one-sided Mann-Whitney-Wilcoxon test was used to assess the differences between patient subgroups stratified by each feature. Our results (Fig 3) showed that the methRisk was significantly correlated (i.e., P < 0.05) with the MELD score (< 8 versus >= 8, P = 0.04), and transplant criteria (1&2 versus 3&4, P = 0.01). There was a minor difference in the methRisk between patient groups stratified by prior AFP (<= 20 ng/ml versus > 20 ng/ml, P = 0.42), largest tumor size (<= 5 cm versus > 5 cm, P = 0.06), tumor number (= 1 versus >= 2, P = 0.20), and BCLC staging (0&A versus B&C, P = 0.06). The observed correlations between the methRisk and existing prognostic factors demonstrate the cfDNA methylation-based risk score’s ability to distinguish between high-risk and low-risk patients.

**Fig 4:**
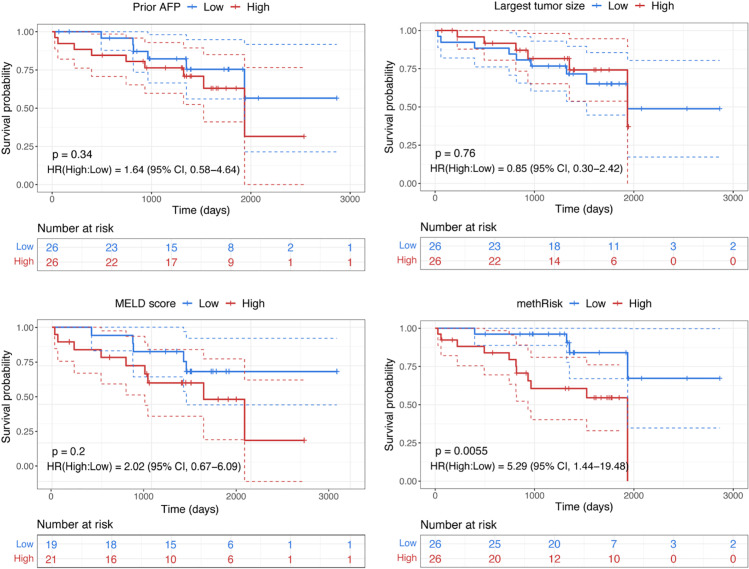
Kaplan-Meier survival curves for overall survival among HCC patient subgroups in the patient cohort with plasma samples. Patients were stratified based on median values of prior AFP, largest tumor size, MELD score, and methRisk. The low-risk group (blue) and high-risk group (red) were compared using a two-sided log-rank test, with the associated p-value indicated. Hazard ratios were calculated via univariate Cox proportional-hazard models.

To evaluate the effectiveness of different features in risk stratification, we generated Kaplan-Meier (KM) survival curves [49] (Fig 4) to illustrate the differences in survival probabilities over time between the patient subgroups in the patient cohort with plasma samples. The patient subgroups were categorized as either low-risk or high-risk based on the median value of features with non-categorical values: prior AFP, largest tumor size, MELD score, and methRisk. Those features with categorical values, such as tumor number and BCLC staging, were excluded from this analysis due to the inability to stratify patients into two groups of roughly equal size, which is important for avoiding biased results [50]. According to the log-rank test [49], the methRisk stratified patients into statistically more distinctive risk groups with higher hazard ratio (HR) and lower p-value (HR = 5.29, 95% CI: 1.44–19.48; P = 0.0055) compared to prior AFP, largest tumor size, and MELD score. Patients with a higher methRisk demonstrated a significantly lower expected survival rate.

**Fig 5:**
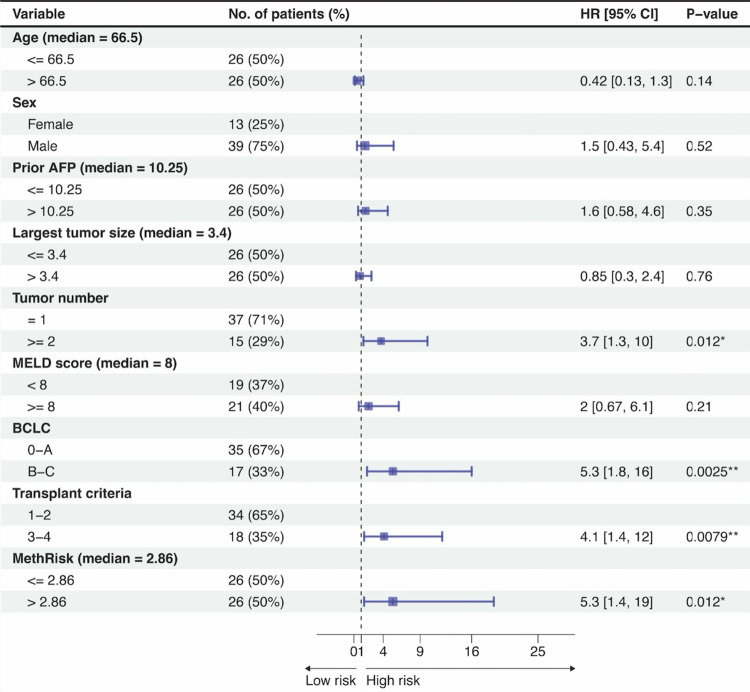
Forest plot displaying the hazard ratios of each feature in univariate analysis in the patient cohort with plasma samples. Patients were stratified into subgroups based on individual features, with the respective group sizes presented. Hazard ratios were estimated using univariate Cox proportional-hazard models and error bars represent the 95% confidence intervals. The p-value is based on the Wald test to assess the significance of the coefficient of a predictor (* P < 0.05, ** P < 0.01).

The univariate Cox analyses (Fig 5) confirmed the significance of methRisk as a risk factor (HR = 5.3, P = 0.012). In the forest plot, patients were categorized into different subgroups according to individual features, and the HRs that compared the risk of death between subgroups were presented. The number of patients in each subgroup is shown in the plot. Note that the only missing values are in the MELD score, which is not available to patients without cirrhosis. In addition to methRisk, tumor number, BCLC stage, and transplant criteria proved to be predictive indicators of OS (P < 0.05). In the next section, we combined the methRisk with these prognostic factors to form an optimal integrated index.

### Enhancing noninvasive prognosis of HCC by integrating methRisk with other prognostic factors

To develop an integrated risk index with optimal performance, we combined the methRisk with BCLC, which demonstrated a strong performance in the univariate analysis (Fig 5). BCLC staging is a noninvasive composite prognostic assessment that integrates imaging, laboratory tests, and clinical information, making it an ideal source for complementing methylation signals. Additionally, we incorporated two critical predictors of HCC patient survival, the normalized values of MELD and AFP, into the composite model to explore the potential of methRisk in enhancing the predictive accuracy of a comprehensive model.

**Fig 6:**
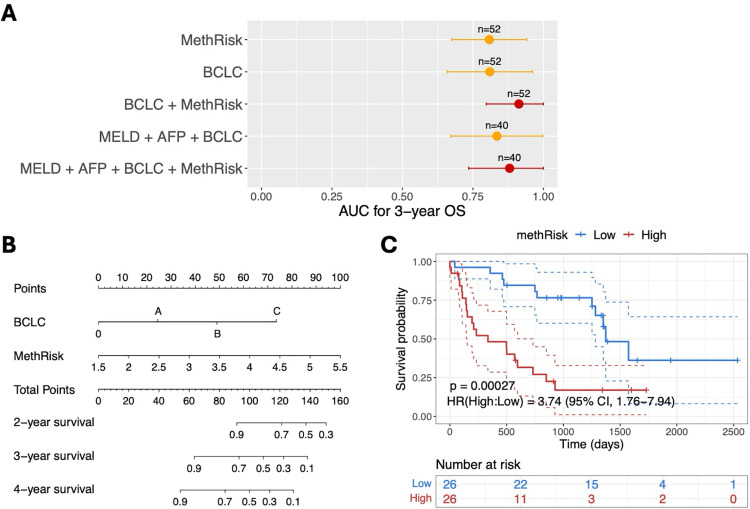
Enhancing noninvasive prognostic prediction with methRisk. **(A)** The performances of features for OS prediction were assessed in the patient cohort with plasma samples. Error bars represent the 95% confidence intervals. The sample size (n) is annotated above the error bar. **(B)** A composite nomogram that integrates BCLC and methRisk for predicting 2-year, 3-year, and 4-year survival. To obtain the predicted survival probability, sum up the points corresponding to each feature and trace a vertical line from the total points to the survival rate scale. **(C)** The Kaplan-Meier survival curve for CCFI between HCC patients stratified by the median value of methRisk. The low-risk group (blue) and high-risk group (red) were compared using a two-sided log-rank test, with the associated p-value indicated. The HR was calculated via a univariate Cox proportional-hazard model.

We computed time-dependent AUCs for the integrated noninvasive predictors in the patient cohort with plasma samples (Fig 6A). The combination of methRisk with other features yielded higher AUCs than using these features alone, indicating that methRisk provides additional prognostic insights distinct from the existing risk indices. Integrating the methRisk with BCLC achieved the best performance, with a 3-year AUC of 0.91 (95% CI = 0.80–1). In the multivariate Cox model, which included both methRisk and BCLC staging, methRisk was an independent risk factor for OS (HR = 2.8, 95% CI: 1.3–5.9; P = 0.007, S4 Table). We also constructed a prognostic nomogram that integrates BCLC and methRisk, based on the multivariate Cox model, to provide a visual and quantitative tool for more accurate risk assessment of patients with HCC (Fig 6B).

Additionally, we evaluated the prognostic performance of AFP, methRisk, and their combination in patients with normal AFP levels (AFP < 10 ng/mL) (S3 Fig). Among these patients, methRisk provides improved stratification, achieving a 3-year AUC of 0.845 (95% CI = 0.672–1), which outperforms AFP (AUC = 0.808, 95% CI = 0.585–1). Combining methRisk with AFP further enhances performance, achieving a 3-year AUC of 0.891 (95% CI = 0.695–1). These results suggest that methRisk captures prognostic information independent of AFP and may serve as a valuable index for risk assessment when AFP alone fails to distinguish between high-risk and low-risk patients.

### Noninvasively predicting cancer-specific survival with methRisk

We also investigated whether the cfDNA-based methRisk can be used to predict an HCC cancer-related survival outcome – composite cancer free interval (CCFI). The CCFI was defined as the time between treatment and recurrence or progression, whichever occurs earlier. We used the methRisk to stratify patients into high- and low-risk groups and plotted a KM curve for CCFI (Fig 6C). Patients with a high methRisk also had a significantly lower cancer-specific survival (HR = 3.74, 95% CI: 1.76–7.94; P = 0.00027). The low-risk group showed a much longer median composite cancer free survival time (~3.5 years) than that of the high-risk group (~1 year). The results suggested that the methRisk can not only predict OS but also predict cancer-specific outcomes, thereby offering clinicians more relevant insights for tailoring treatment strategies.

## Discussion

Noninvasively prognosticating outcomes in HCC patients is a challenging task due to the tumor heterogeneity and the lack of an ideal prognostic biomarker. Despite numerous existing radiological and serum biomarkers utilized in clinical practice, current staging systems still poorly discriminate survival outcomes in patients who appear to have a similar disease burden. This has particular relevance and impact when it comes to selecting patients for curative therapy, such as liver transplantation, where decisions regarding scarce resource allocation are predicated on achieving acceptable post-transplant oncologic outcomes. Recent studies have demonstrated that cfDNA harbors genetic and epigenetic alterations relevant to cancer development and progression, making it a promising candidate for noninvasive cancer prognosis. Therefore, we aim to investigate the potential of cfDNA methylation analysis in the noninvasive prognostication of HCC via a simple peripheral blood draw.

We identified 158 methylation markers through a rigorous selection process utilizing a large dataset of tissue samples from TCGA. These markers were validated in an independent HCC tumor tissue cohort, confirming their high prognostic value. Functional and transcriptomic analyses of genes related to the methylation markers further suggest that they may contribute to cancer progression and affect HCC prognosis. We presented top enriched pathways as illustrative examples. Our epigenetic signature involves genes related to nucleotide metabolism, especially in purine metabolism, such as GMPR2, TAF9, ENTPD4, and RRM2B. These genes are important for cellular proliferation and DNA repair, which are crucial in cancer development. The analysis also pointed to genes such as TSC2, PLD2, and DGKQ, which are associated with the choline metabolism and the phospholipase D signaling pathway. These pathways regulate cell growth, survival, and migration, highlighting their potential impacts on cancer progression. Moreover, we identified genes involved in lysosome function, including AP3D1, MFSD8, IGF2R, and ENTPD4. Lysosomes are essential for cellular homeostasis and can influence tumor progression through altered metabolism and immune response. Additionally, genes such as TSC2, FAM134C, USP36, TECPR1, and LRSAM1 are involved in pathways including macroautophagy and selective autophagy, which can contribute to cancer when dysregulated. Our marker set also encompasses the MCM7 gene, which is relevant to repairing double-strand DNA breaks and preventing genomic instability in cancers.

The value of DNA methylation profiles from liver tissues in predicting outcomes for HCC patients has been highlighted in previous research. These investigations have identified methylation markers in tumor tissues and in cirrhotic tissues adjacent to tumors that can be indicative of patient outcomes [6,7]. However, a significant limitation of relying on tissue-based methylation markers is the requirement for invasive biopsy procedures. In addition, obtaining a biopsy from a small area of a tumor via percutaneous biopsy may not reflect the complete genetic and epigenetic landscape of the entire tumor, which could result in prognostic inaccuracies [3]. In contrast, cfDNA is shed by all areas of the tumor into the bloodstream, therefore it offers a more comprehensive view of the tumor’s methylation profile. Additionally, predicting cfDNA-based methRisk from a blood draw presents a significant advantage in terms of convenience and patient comfort, enabling both earlier and repeated risk assessments and interventions that could play a pivotal role in improving patient outcomes.

Previous studies have explored the use of limited cfDNA methylation marker panels for HCC prognosis [8]. However, these small panels do not adequately represent the extensive range of epigenetic aberrations linked to HCC and lack the sensitivity to accurately capture reliable cancer signals in the plasma. In contrast, our study adopted a recently developed cost-effective cfDNA-methylome sequencing technique (i.e., cfMethyl-Seq), allowing us to systematically examine a large panel of cfDNA methylation markers for the prognostication of survival in HCC patients. Furthermore, our HCC prognostic model showed great potential for survival prediction and patient stratification. The results indicate that cfDNA-based methRisk provides prognostic information orthogonal to that of existing composite risk indices such as BCLC. The integrated index of methRisk and BCLC outperformed all the other features, suggesting that methRisk effectively enhances the predictive accuracy of existing noninvasive composite prognostic assessments. Additionally, we demonstrated that methRisk has the dual capability of predicting not only OS but also cancer-specific outcomes, which can be more clinically relevant for guiding personalized treatment strategies.

We recognize that there are certain limitations in our study. First, the small sample size of the plasma cohort and the presence of censoring in the survival data potentially diminishes the statistical power of the analysis. To address this limitation, future work could focus on larger validation studies and external replication to establish the robustness and generalizability of our findings across diverse patient populations. Leveraging larger and more diverse datasets will help refine our prognostic model, improving its predictive accuracy and clinical applicability. Multi-center collaborations will further enhance the reliability and reproducibility of our approach, strengthening its potential for clinical translation. Furthermore, to improve the predictive performance of the prognostic model, particularly in different patient populations, additional investigations could be directed towards the development of calibration algorithms. Lastly, we acknowledge that the diverse nature of cancer pathogenesis means that a single model may not adequately predict risk scores across all clinical scenarios. It would be beneficial to develop specialized models tailored to different HCC etiologies or specific clinical characteristics to provide more targeted and effective prognostic tools.

In summary, we have systematically identified a large panel of cfDNA methylation biomarkers for HCC prognosis and validated their predictive accuracy in plasma samples. The cfDNA-based methRisk, derived from these circulating biomarkers, effectively stratifies patients into clinically relevant risk groups and predicts patient survival with high accuracy. Incorporating cfDNA methylation analysis into the existing noninvasive prognostic framework for HCC patients holds great potential for enhancing the effectiveness of current staging systems and optimizing patient selection for curative therapies like liver transplantation.

## Supporting information

S1 File Data preprocessing. (DOCX)

S1 Fig Heatmaps of the top 30 methylation markers with the highest C-index in tissue and plasma cohorts. (A) Methylation status in the patient cohort with tissue samples. (B) Methylation status in the patient cohort with plasma samples. Samples (columns) are sorted by risk score. The marker index name and the concordance index (C-index) for each marker are annotated in the heatmap rows. Higher C-index values indicating stronger predictive power for survival.
(TIF)

S2 Fig Time-dependent AUCs for individual features predicting 3-year survival in the cohort with tissue samples. Error bars represent the 95% confidence intervals. The sample size (n) is annotated to the right of the error bar.
(TIF)

S3 Fig Performance of AFP, methRisk, and their combination in OS prediction for patients with normal AFP levels (AFP < 10 ng/mL) in the plasma cohort. Error bars represent the 95% confidence intervals.
(TIF)

S1 Table Characteristics of 29 HCC patients with tissue samples. (DOCX)

S2 Table Detailed information on methylation markers. (CSV)

S3 Table Transcriptomics analyses of markers association with survival. (CSV)

S4 Table Multivariate Cox regression model including cfDNA-based methRisk and BCLC. (DOCX)

## Abbreviations

AFP: alpha-fetoprotein
ALD: alcoholic liver disease
AUC: area under the time-dependent receiver operating characteristic curve
BCLC: Barcelona Clinic Liver Cancer
CCFI: composite cancer free interval
cfDNA: cell-free DNA
Coxnet: Cox model regularized by elastic net penalties
CpG: 5’—C—phosphate—G—3’
DMP: differentially methylated probe
DNMIVD: DNA Methylation Interactive Visualization Database
GENCODE: Genome as Encyclopedia of DNA Elements
GO: Gene Ontology
HBV: hepatitis B virus
HCC: hepatocellular carcinoma
HCV: hepatitis C virus
HR: hazard ratio
IQR: interquartile range
KEGG: Kyoto Encyclopedia of Genes and Genomes
KM: Kaplan-Meier
LRT: locoregional treatment
MASH: metabolic dysfunction-associated steatohepatitis
MASLD: metabolic dysfunction-associated steatotic liver disease
MELD: model for end-stage liver disease
methRisk: methylation-based overall survival risk score
OS: overall survival
RRBS: Reduced Representation Bisulfite Sequencing
RSF: random survival forest
SNP: single-nucleotide polymorphism
ST: systemic therapy
TCGA: The Cancer Genome Atlas
UCLA: University of California, Los Angeles
UCSF: University of California, San Francisco
UMI: unique molecular identifier
