# Supplementary material for: Noninvasive prognostication of hepatocellular carcinoma based on cell-free DNA methylation
Source: PLoS One. 2025 Apr 25;20(4):e0321736. doi: 10.1371/journal.pone.0321736 (PMC12026916; doi:10.1371/journal.pone.0321736)
Supplement: S1 File — (DOCX) [file pone.0321736.s001.docx]

**S1 File. Data preprocessing.**

cfMethyl-Seq libraries were generated for 52 cfDNA samples and were sequenced with 150 bp paired-end reads on Illumina machines by Genewiz, Inc. (South Plainﬁeld, NJ, USA). The cfMethyl-Seq library construction was conducted as previously reported. We performed three steps to preprocess the cfMethyl-Seq data. In Step 1, the unique molecular identifier (UMI) sequence was removed and the read was trimmed. Our custom adapters contain an 8 bp random UMI and a 5 bp ﬁxed sequence at the beginnings of both forward and reverse reads. These sequences are removed before adapter trimming (and written into the read name). Then Trim-galore [1] was used to trim the default Illumina adapters from the sequencing reads (using the options -three_prime_clip_R1 15 --three_prime_clip_R2 13 --clip_R2 2 --length 15 --phred33). In Step 2, we performed sequence alignment, deduplication and methylation calling. We ﬁrst used Bismark [2] to align the trimmed reads to the reference genome hg19 (GRCh37 (GCA 000001405.1)). Then Umi-Grinder [3] was used to remove PCR duplicates based on the UMI labels (now in the read names), allowing 4 mismatches in the total 16 bp UMI. Bismark [2] methylation extractor was then used to call methylation in the mapped, deduplicated reads. In Step 3, the mapping locations of R1 and R2 were merged to form one fragment. Tissue RRBS samples were sequenced and processed in the same manner as cfMethyl-Seq data.

**References**

1. Krueger F. Software “Trim Galore.” https://www.bioinformatics.babraham.ac.uk/projects/trim_galore/.

2. Krueger F, Andrews SR. Bismark: a flexible aligner and methylation caller for Bisulfite-Seq applications. Bioinformatics. 2011;27:1571–1572.

3. Krueger F. Unique Molecule Identiﬁers (UMIs) based sequencing deduplication software. https://github.com/FelixKrueger/UmiGrinder.
