# Supplementary figures and images for: Noninvasive prognostication of hepatocellular carcinoma based on cell-free DNA methylation
Source: PLoS One. 2025 Apr 25;20(4):e0321736. doi: 10.1371/journal.pone.0321736 (PMC12026916; doi:10.1371/journal.pone.0321736)

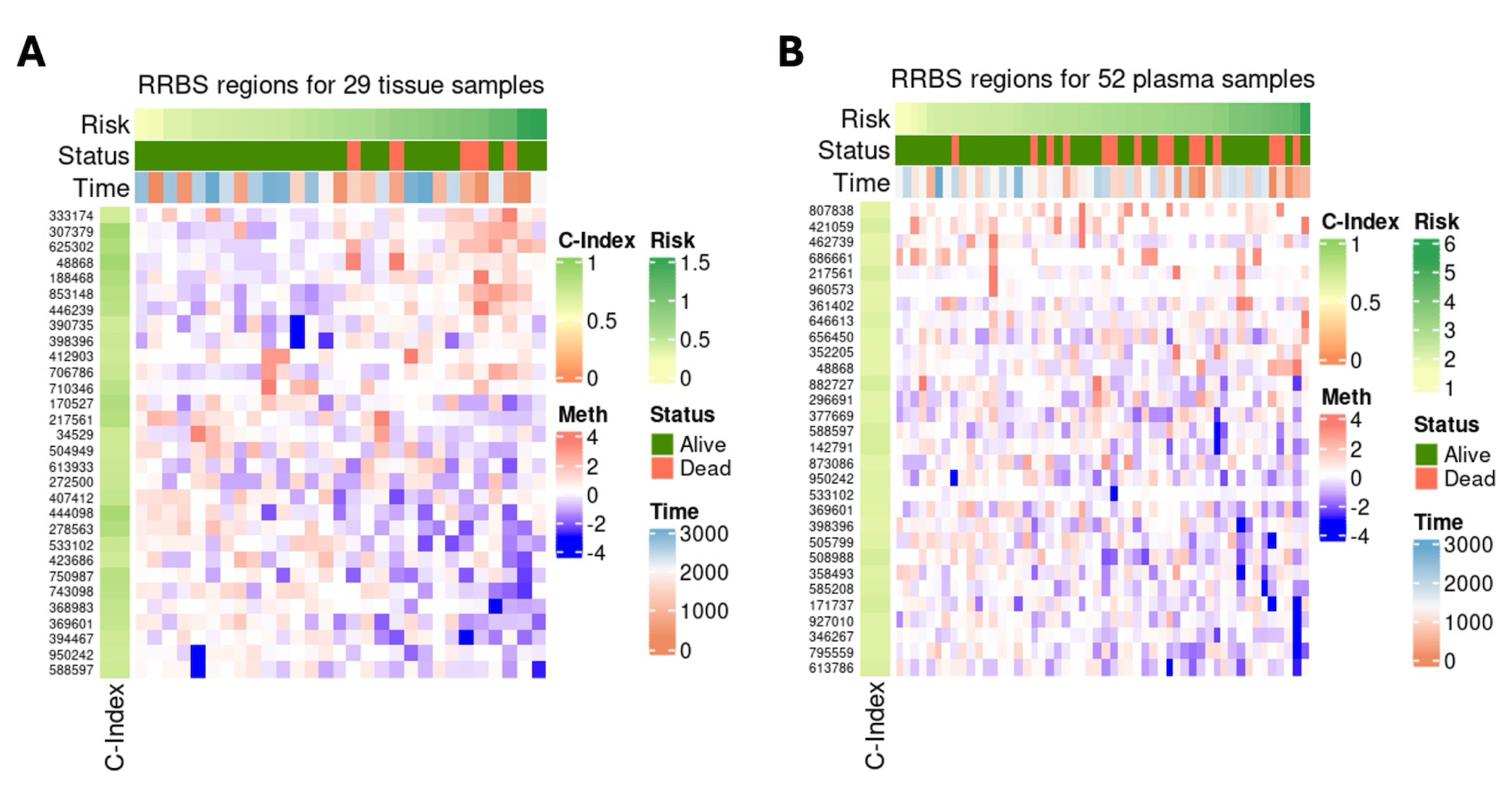

Supplement: S1 Fig — (A) Methylation status in the patient cohort with tissue samples. (B) Methylation status in the patient cohort with plasma samples. Samples (columns) are sorted by risk score. The marker index name and the concordance index (C-index) for each marker are annotated in the heatmap rows. Higher C-index values indicating stronger predictive power for survival. (TIF) [file pone.0321736.s002.tif]

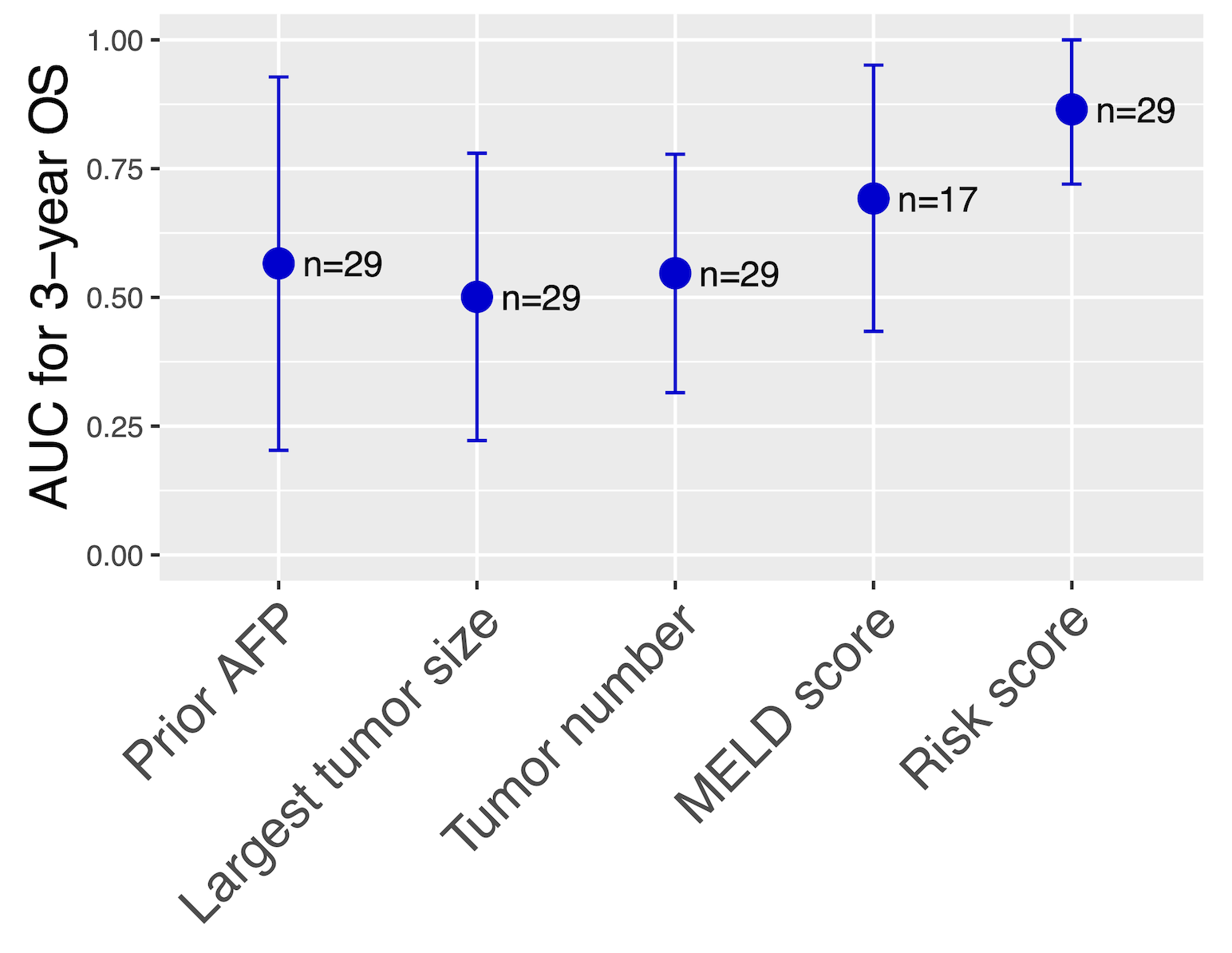

Supplement: S2 Fig — Error bars represent the 95% confidence intervals. The sample size (n) is annotated to the right of the error bar. (TIF) [file pone.0321736.s003.tif]

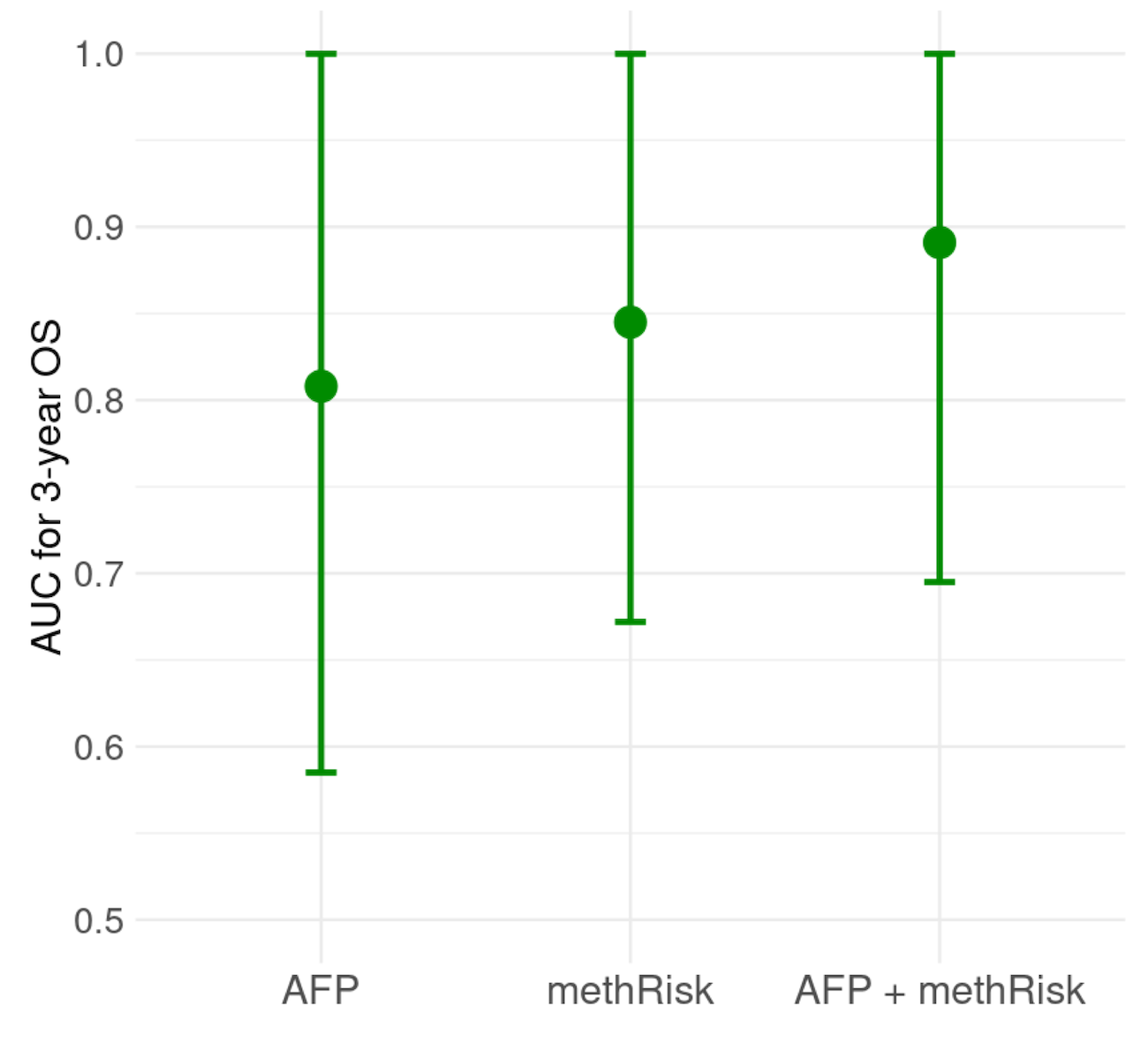

Supplement: S3 Fig — Error bars represent the 95% confidence intervals. (TIF) [file pone.0321736.s004.tif]
