# Supplementary material for: Noninvasive prognostication of hepatocellular carcinoma based on cell-free DNA methylation
Source: PLoS One. 2025 Apr 25;20(4):e0321736. doi: 10.1371/journal.pone.0321736 (PMC12026916; doi:10.1371/journal.pone.0321736)
Supplement: S1 Table — (DOCX) [file pone.0321736.s005.docx]

**S1 Table. Characteristics of 29 HCC patients with tissue samples.**

| **Characteristic** | **No.** |
| --- | --- |
| Median age (IQR) | 65 (60, 69) |
| Sex, male (%) | 23 (79.3%) |
| Etiology (%)  HCV  HBV  MASH  ALD  Others | 17 (58.6%)  9 (31%)  0 (0%)  0 (0%)  3 (10.3%) |
| Median largest tumor size, cm (IQR) | 3.2 (2.3, 5.0) |
| Multiple nodules present (%) | 4 (13.8%) |
| Median prior AFP, ng/ml (IQR) | 23.6 (4, 222) |
| Median MELD score (IQR) | 8 (7, 12) |
| BCLC stage (%)  0  A  B  C | 0 (0%)  24 (82.8%)  3 (10.3%)  2 (6.9%) |
| Transplant criteria  1  2  3  4 | 21 (72.4%)  3 (10.3%)  5 (17.2%)  0 (0%) |
| LRT and/or ST before sample collection, yes (%) | 7 (24.1%) |
| LRT and/or ST after sample collection, yes (%) | 8 (27.6%) |
| Events (%)  Death  Recurrence or progression | 5 (17.2%)  15 (51.7%) |
| Median follow-up, months | 67 |

Abbreviation: AFP, alpha-fetoprotein; ALD, alcoholic liver disease; BCLC, Barcelona Clinic Liver Cancer; HBV, hepatitis B virus; HCV, hepatitis C virus; IQR, interquartile range; LRT, locoregional treatment; MASH, metabolic dysfunction-associated steatohepatitis; MELD, model for end-stage liver disease; ST, systemic therapy.
