# Supplementary material for: Noninvasive prognostication of hepatocellular carcinoma based on cell-free DNA methylation
Source: PLoS One. 2025 Apr 25;20(4):e0321736. doi: 10.1371/journal.pone.0321736 (PMC12026916; doi:10.1371/journal.pone.0321736)
Supplement: S4 Table — (DOCX) [file pone.0321736.s008.docx]

**S4 Table. Multivariate Cox regression model** **including cfDNA-based methRisk and BCLC.**

|  | Coeﬃcients | HR | CI | P-value |
| --- | --- | --- | --- | --- |
| BCLC | 1.01 | 2.74 | [1.51, 4.98] | 0.0009 |
| methRisk | 1.03 | 2.80 | [1.32, 5.94] | 0.007 |
